# Non-Hodgkin Lymphoma Risk and Variants in Genes Controlling Lymphocyte Development

**DOI:** 10.1371/journal.pone.0075170

**Published:** 2013-09-30

**Authors:** Johanna M. Schuetz, Denise Daley, Stephen Leach, Lucia Conde, Brian R. Berry, Richard P. Gallagher, Joseph M. Connors, Randy D. Gascoyne, Paige M. Bracci, Christine F. Skibola, John J. Spinelli, Angela R Brooks-Wilson

**Affiliations:** 1 Canada’s Michael Smith Genome Sciences Centre, BC Cancer Agency, Vancouver, British Columbia, Canada; 2 Faculty of Medicine, University of British Columbia, Vancouver, British Columbia, Canada; 3 Department of Epidemiology, Comprehensive Cancer Center, University of Alabama at Birmingham, Birmingham, Alabama, United States of America; 4 Department of Pathology, Royal Jubilee Hospital, Victoria, British Columbia, Canada; 5 Cancer Control Research, BC Cancer Agency, Vancouver, British Columbia, Canada; 6 Division of Medical Oncology and Centre for Lymphoid Cancer, BC Cancer Agency, Vancouver, British Columbia, Canada; 7 Department of Pathology and Centre for Lymphoid Cancer, BC Cancer Agency, Vancouver, British Columbia, Canada; 8 Department of Epidemiology and Biostatistics, University of California San Francisco, San Francisco, California, United States of America; 9 School of Population and Public Health, University of British Columbia, Vancouver, British Columbia, Canada; 10 Department of Biomedical Physiology and Kinesiology, Simon Fraser University, Burnaby, British Columbia, Canada; University of North Carolina at Chapel Hill, United States of America

## Abstract

Non-Hodgkin lymphomas (NHL) are a heterogeneous group of solid tumours of lymphoid cell origin. Three important aspects of lymphocyte development include immunity and inflammation, DNA repair, and programmed cell death. We have used a previously established case-control study of NHL to ask whether genetic variation in genes involved in these three important processes influences risk of this cancer. 118 genes in these three categories were tagged with single nucleotide polymorphisms (SNPs), which were tested for association with NHL and its subtypes. The main analysis used logistic regression (additive model) to estimate odds ratios in European-ancestry cases and controls. 599 SNPs and 1116 samples (569 cases and 547 controls) passed quality control measures and were included in analyses. Following multiple-testing correction, one SNP in *MSH3*, a mismatch repair gene, showed an association with diffuse large B-cell lymphoma (OR: 1.91; 95% CI: 1.41–2.59; uncorrected *p* = 0.00003; corrected *p* = 0.010). This association was not replicated in an independent European-ancestry sample set of 251 diffuse large B-cell lymphoma cases and 737 controls, indicating this result was likely a false positive. It is likely that moderate sample size, inter-subtype and other genetic heterogeneity**,** and small true effect sizes account for the lack of replicable findings.

## Introduction

Non-Hodgkin lymphoma (NHL) is a collection of malignancies of lymphocyte origin. In Western countries, 85% of NHLs have a B-cell origin. NHL subtypes vary in prognosis, treatment options and outcome. Diffuse large B-cell lymphoma (DLBCL) patients with different molecular or genetic abnormalities can have diverse presentation and outcomes. Risk of developing NHL can be influenced by both environmental and genetic factors that affect the survival of lymphocytes.

Lymphocyte development is a complex process, with checkpoints in place to ensure that the cells whose function is to quickly and effectively protect the host from a variety of offences, will also withhold such an assault on host cells. Cell growth and cell death need to be regulated so that the number of lymphocytes is controlled in such a way that they are sufficient to fight infections, but not so numerous that they are a burden to maintain. Three important aspects of this control are: 1) immunity and inflammation to respond to stimuli that cause their activation and rapid cell cycle division; 2) DNA repair to counteract errors from cell division or lymphocyte receptor gene rearrangement; and 3) cell death to remove lymphocytes that are not able to meet cell cycle checkpoints and/or reduce autoimmunity.

Previous work by several research groups has identified genetic variants associated with NHL in genes related to B-cell survival [1], [2], DNA repair [3] and immunity and inflammation[4]–[8]. Collectively, genetic variants in these types of genes are likely to play a role in susceptibility to NHL. To survey for genetic factors associated with NHL in genes involved in immunity and inflammation, DNA repair or cell death, we selected 118 genes (listed in **Table S1 in File S1**) related to these biological processes, tagged them with SNPs and tested them for association with NHL in 569 cases and 547 controls. In addition, we selected 39 SNPs that had previously been associated with NHL in the literature, and tested them for replication in our study. After correction for multiple testing, we found evidence that a SNP in *MSH3*, a gene that has never before been implicated in NHL, may affect susceptibility to DLBCL; however, this association did not replicate in an independent NHL population.

## Materials and Methods

The samples and genes tested in this study were part of a 1536-SNP Illumina GoldenGate panel that included SNPs from candidate genes related to other pathways and hypotheses [9]. Details of the population, samples and methodology have been previously described [10].

### Study Subjects and Samples

All new NHL cases in the Greater Vancouver Regional District and Greater Victoria (Capital Regional District), British Columbia, from March 2000 to February 2004 were invited to participate. Cases aged 20 to 79 were included. Patients with prior transplant or HIV-positivity were excluded. Population controls were frequency matched by age (within 5-year groups), sex and area of residence. Family history of cancer was based on subject-reported data. Of 821 cases and 848 controls were available for this study, 797 cases and 790 controls had sufficient DNA for genotyping. The study was approved by the joint University of British Columbia/British Columbia Cancer Agency Research Ethics Board; all participants gave written informed consent.

DNA was extracted from whole blood (407 samples), lymphocytes isolated from blood (782 samples), mouthwash (24 samples), or saliva (48 samples) as previously described [9]. 326/1587 samples, referred to as ‘WGA samples’, had low DNA yields; their DNA was amplified by whole genome amplification using the RepliG kit (QIAGEN, Mississauga, ON, Canada) [9].

### Genotyping

The 118 genes selected for this study (**Table 1**) were based on a review of the biological literature. For each gene, publicly available data from HapMap phase II was imported into Haploview [11] for tagSNP selection using Tagger at *r* ^2^ = 0.8. TagSNP selection was restricted to SNPs with minor allele frequency (MAF) >5%. In addition, 39 specific SNPs previously reported as associated with NHL, autoimmune disease or cancer were included to test for replication of these associations in our study. These ‘replication’ SNPs are listed in **Table S2 in File S1**. 51 ancestry-informative markers (AIMs) selected from Halder *et al*. [12] were also included in the assay. Genotyping was done using the Golden Gate system (Illumina, San Diego, CA), at The Centre for Applied Genomics, the Hospital for Sick Children in Toronto, Canada; as described previously [9].

**Table 1:** Genes and categories.

Immunity and Inflammation	*AICDA, BRD2, CCL5, CD69, CD74, CD81, CTLA4, HFE, IFNAR2, IFNB1, IFNG, IL10RA, IL1RN, IL4, IL6, IL7, IL7R, IRF4, IRF5, ITGAM, JAK1, JAK3, KIAA1542, LTA, PKX, PRDM1, PRMT5, SPIB, SPP1, STAT3*
Cell death	*AGTR1, APAF1, BAD, BAK1, BCL11A, BCL2L1, BCL2L2, BID, BIK, BIRC3, BMF, CASP1, CASP10, CASP3, CASP4, CASP8, CD40, CDH22, CFLAR, FASLG, IGFBP3, IL2, IL8, IL8RB, ITCH, MDM4, MYC, NEDD4, NFKB1, NFKB2, PARP1, PAX5, RASSF1, REL, RELA, RELB, TLR2, TNFSF10, TP73, ZFX*
DNA repair	*APEX1, ATR, BIN3, C11orf30, CCND1, CDK7, CDKN2A, CHEK1, CHEK2, E2F1, E2F2, E2F3, ERCC2, ERCC5, EXO1, H2AFZ, HIC1, HINT1, LIG1, LIG3, LIG4, MGMT, MSH2, MSH3, MSH6, MTHFR, MTR, OGG1, PLK1, PMS2, POLB, POLD1, PTEN, RAD51, RAD52, RAD54B, RAG1, RB1, RPA1, TP53BP1, TYMS, UNG, WRN, XRCC1, XRCC3, XRCC4, XRCC5, YY1*

Quality control (Q/C) was conducted using Genome Studio version 2009.1 (Illumina, San Diego, CA) and systems and databases developed in the laboratory of DD [13]. Genotypes derived from WGA DNA and genomic DNA were subjected to Q/C separately. 1411 samples (717 cases and 694 controls) passed Q/C (**Table 2**); 1116/1411 samples (569 cases and 547 controls) were of European ancestry and subsequently included in statistical analysis [9]. AIMS analysis in this study has been previously described [9], and supported analysis of the European-ancestry samples as one group.

**Table 2:** Samples that passed Q/C.

	Controls (%)	Cases (%)
**Pathology**		
** B-cell lymphomas**		
DLBCL	–	189 (26%)
FL	–	205 (29%)
MZL/MALT	–	78 (11%)
MCL	–	43 (6%)
SLL/CLL	–	39 (5%)
LPL	–	40 (6%)
MISC BCL	–	54 (8%)
** T-cell lymphomas**		
MF	–	38 (5%)
PTCL	–	24 (3%)
MISC TCL	–	7 (1%)
**Ethnicity**		
Caucasian	547 (79%)	569 (79%)
Asian	69 (10%)	66 (9%)
South Asian	31 (4%)	26 (4%)
Mixed/Other	29 (4%)	33 (5%)
Refused/Unknown	18 (2%)	23 (3%)
**Gender**		
Male	360 (52%)	416 (58%)
Female	334 (48%)	301 (42%)
**Age group (years)**		
20–49	172 (25%)	131 (18%)
50–59	153 (22%)	173 (24%)
60–69	185 (27%)	196 (27%)
70+	184 (27%)	217 (30%)
**Total**	694 (100%)	717 (100%)

DLBCL = Diffuse Large B-Cell Lymphoma, FL = Follicular Lymphoma, MZ/MALT = Marginal Zone lymphoma/Mucosa-Associated Lymphoma Tissue lymphoma,MCL = Mantle Cell lymphoma, SLL = Small Lymphocytic Lymphoma, LPL = Lymphoplasmacytic Lymphoma, Misc. B-cell = Miscellaneous B-cell lymphoma, MF = Mycosis Fungoides, PTCL = Peripheral T-Cell Lymphoma, Misc. T-cell = Miscellaneous T-cell lymphoma.

Of 708 SNPs selected for genotyping of variants in genes related to lymphocyte development, 109 were excluded at the genotype Q/C stage (32 SNPs were rejected by the genotyping centre upon initial inspection, 14 for low GenTrain scores, 26 for being potential copy number variants, 12 for being monoallelic, 8 for having a call rate <0.95, 15 for having any error between duplicate genotypes, and 2 for deviating significantly from Hardy-Weinberg equilibrium [HWE]). An additional 160 SNPs failed Q/C only in WGA samples (8 upon initial inspection by the genotyping centre, 49 for low GenTrain score, 64 for call rate <0.95, 38 SNPs that had discrepant genotypes between WGA samples and pre-WGA matched DNA, and 1 SNP for being out of HWE), and 4 SNPs failed Q/C only in mouthwash or saliva samples. This left 599 SNPs (85%), listed in **Table S3 in File S1**, for analysis in all non-WGA samples and 439 SNPs in both blood and WGA samples.

### Statistical Analysis

Statistical analyses were conducted in SVS Suite 7 (Golden Helix, Bozeman, MT). Logistic regression (additive model) was fit for diffuse large B-cell lymphoma (DLBCL), follicular lymphoma (FL), marginal zone lymphoma (MZL), all B-cell NHLs and all T-cell NHLs. Other NHL subtypes were not individually tested, as sample numbers were insufficient. In all subtype analyses, selected cases were compared to all controls. The analysis was restricted to European-ancestry samples, with other ethnicities (Asian, south-east Asian and “other”) only tested when SNPs showed association in European-ancestry samples, corresponding to 148 DLBCL, 165 FL, 55 MZL, 523 B-cell NHL, 45 T-cell NHL and 547 control samples. This corresponded to a minimum detectable odds ratio of 1.54 for DLBCL, 1.51 for FL, 1.88 for MZL, 1.33 for B-cell NHL and 1.99 for T-cell NHL. For each SNP, *p-*values were calculated for the model with the SNP of interest vs. the basic model (which accounted for 5-year age groups, sex, and region). For only the SNPs that showed a statistically significant association, to find the model with the best fit we then tested dominant and recessive models in genotypic tests using the chi-squared test, as well as a recessive model by logistic regression with the adjustments listed above (i.e. age groups, sex and region). SNPs that showed an association were also tested for interaction with sex by comparing a model including the SNP, age group, sex and region to a model that also included the SNP*sex interaction. In genes that contained multiple SNPs with an association, the SNPs that showed an association were tested for interaction by comparing a model including that included the two SNPs, age group, sex and region vs. a model with the addition of the SNP*SNP interaction. In addition, for genes with an association, haplotype analysis was conducted in SVS Suite 7.

To correct for multiple testing, we have used a two-tiered approach, as previously described [9]. The Benjamini-Hochberg procedure [14], implemented in R version 2.11.1, was applied to control the false-discovery rate (FDR) for SNPs within each gene, giving a corrected *p*-value denoted as *p*_G_. The smallest adjusted *p*-value for each gene was taken to represent the gene, and FDR was applied again across the genes in each of the three hypotheses (i.e. gene categories) tested (cell death, DNA repair and immunity and inflammation). This second corrected *p*-value was denoted *p_H_*. Adjusted *p*-values <0.05 were considered statistically significant. No multiple-testing correction was applied for the few interaction or haplotype tests.

Since genes involved in mismatch repair pathways have been shown to be important for colorectal cancer risk, we tested whether rs33003 in *MSH3* were associated with a family history of colorectal cancer. Colorectal cancer in one or more first-degree relatives of the NHL cases and controls was coded as a true/false “family history of colorectal cancer” variable, and was used in logistic regression analysis in European-ancestry samples, adjusting for sex, region and 5-year age groups. 28/569 cases and 33/547 controls of European-ancestry had a family history of colorectal cancer.

### Replication

The association of rs33003 with DLBCL was tested in a previously described independent population from the San Francisco Bay Area [15]. Briefly, cases were identified through the Northern California Cancer Center between 2001 and 2005. All were residents of the San Francisco Bay Area, 20–84 years old, and provided informed consent. For this analysis, we used genotypes imputed by BEAGLE v.3.3 [16] for 737 controls and 251 DLBCL cases that self-reported as “non-Hispanic white” and also clustered with Caucasian samples by principal component analysis. The imputation yielded 391 samples of GG genotype, 417 samples of GA genotype, 103 samples of AA genotype and 77 samples with unknown genotype. A logistic regression model under the additive model, with correction for age and sex was used to estimate odds ratios.

## Results

**Figure 1:**
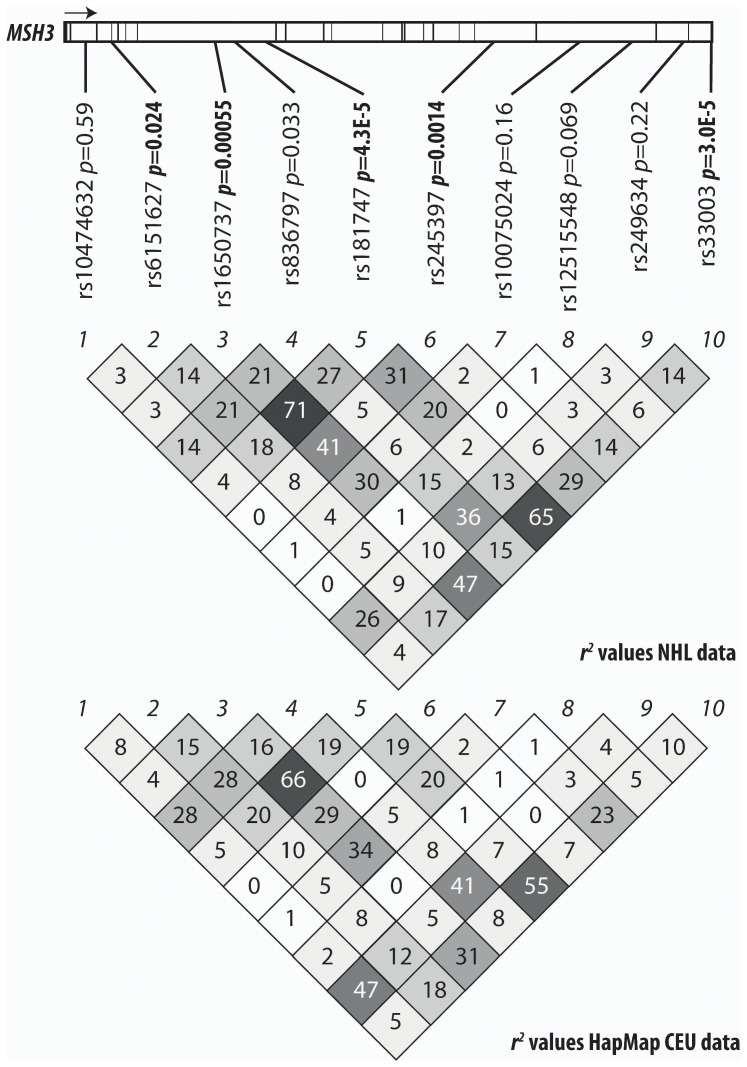
Association results and linkage disequilibrium in *MSH3*. *r* ^2^ values for our genotyped samples are shown in the top section (“*r^2^* values in NHL data”) and *r* ^2^ from the CEU population of HapMap are shown in the bottom section (“*r^2^* values in HapMap CEU data”). The gene model of *MSH3* is shown on top, 5′ to 3′ from left to right, with vertical lines marking exons. *p*-values (before correction for multiple testing) are from the analysis in DLBCL samples of European ancestry.

**Table S4 in File S1** lists all SNPs with *p*<0.05 (before any multiple testing correction). **Table 3** lists the 59 SNPs with *p_G_*<0.05. Of note, none of the 39 SNPs selected to replicate previously reported associations were associated with lymphoma in our population. Only one SNP showed an association that was significant after multiple testing correction both at the individual gene and multi-gene (hypothesis) level. rs33003, located in *MSH3*, was significantly associated with DLBCL (OR per allele: 1.91 [95% CI: 1.41–2.59]; *p*_G_ = 0.0002; *p*_H_ = 0.0103). It is a common SNP, with MAF 0.32. We found the recessive model best fits the inheritance mode of rs33003 (**Table S5 in File S1**). Many SNPs in the same region had low *p*-values in the analysis with DLBCL (**Figure 1**). The second most strongly associated SNP in *MSH3*, rs181747, is in moderate linkage disequilibrium with rs33003, with *r* ^2^ = 0.55 in HapMap data and *r^2^* = 0.65 in our data set. There is evidence for an interaction between these two SNPs (*p* = 0.0014). However, no haplotype of SNPs in this region was more strongly associated with DLBCL than either of these two SNPs alone. There was no statistically-significant association of rs33003 or rs181747 with DLBCL in 21 cases and 69 controls of Asian descent (OR: 0.68 [95% CI: 0.28–1.64], *p*_G_ = 1.00; and OR: 0.94 [95% CI: 0.45–1.93], *p*_G_ = 1.00, respectively) or 6 cases and 31 controls of South-Asian ancestry descent (OR: 1.86 [95% CI: 0.45–7.61], *p*_G_ = 0.5483; and OR: 2.43 [95% CI: 0.62–9.50], *p*_G_ = 0.4849, respectively); the number of samples in these groups is too small to make a statement about associations in these groups. There was also no evidence for interaction between rs33003 and rs181747 in Asian ancestry samples (*p* = 0.1957) or South-Asian ancestry samples (*p* = 0.9873).

**Table 3:** Logistic regression analysis results for SNPs with *p_G_*<0.05.

Subtype	Category	Gene	SNP	Coordinates*	Alleles	Odds ratio	95% CI	*p*-value	*p_G_*	*p_H_*
BCL	Cell death	*TP73*	rs3765703	1:3592436	T/G	0.72	0.61–0.86	0.00032	0.00443	0.17282
		*TP73*	rs3765702	1:3592187	C/T	0.71	0.58–0.86	0.00047	0.00443	–
		*TP73*	rs1885859	1:3583692	C/G	0.76	0.64–0.91	0.00249	0.01577	–
		*NEDD4*	rs11630780	15:56128445	A/C	1.29	1.08–1.53	0.00390	0.03120	0.40560
		*CASP10*	rs12693932	2:202093395	T/C	1.23	1.04–1.46	0.01461	0.01461	0.28494
	DNA repair	*MSH3*	rs181747	5:80016874	T/C	1.36	1.13–1.64	0.00128	0.01277	0.19655
		*E2F2*	rs3218203	1:23837560	C/G	1.41	1.13–1.76	0.00236	0.01419	0.19655
		*APEX1*	rs1130409	14:20925154	G/T	0.76	0.64–0.91	0.00282	0.00282	0.13525
		*E2F3*	rs10946384	6:20495546	C/T	1.40	1.12–1.74	0.00283	0.04528	0.40187
		*MSH3*	rs33003	5:80171134	G/A	1.36	1.11–1.67	0.00287	0.01433	–
		*MSH3*	rs245397	5:80101773	C/T	1.38	1.11–1.73	0.00430	0.01433	–
		*RB1*	rs4151510	13:48945175	G/A	0.68	0.52–0.89	0.00546	0.01638	0.19655
		*MSH3*	rs1650737	5:80001785	A/G	1.30	1.07–1.58	0.00801	0.01771	–
		*MSH3*	rs6151627	5:79965536	A/G	0.77	0.63–0.94	0.00886	0.01771	–
DLBCL	Cell death	*TP73*	rs3765703	1:3592436	T/G	0.66	0.51–0.87	0.00272	0.03023	0.83626
		*TP73*	rs3765702	1:3592187	C/T	0.64	0.48–0.87	0.00318	0.03023	–
	Inflammation & Immunity	*LTA*	rs2844484	6:31536224	G/A	0.65	0.49–0.86	0.00183	0.01466	0.43980
		*IL7R*	rs1494571	5:35880087	G/C	0.66	0.48–0.90	0.00700	0.03502	0.52530
		*LTA*	rs2239704	6:31540141	C/A	0.69	0.52–0.92	0.00908	0.03634	–
	DNA repair	*MSH3*	rs33003	5:80171134	G/A	1.91	1.41–2.59	0.00003	0.00022	***0.01032***
		*MSH3*	rs181747	5:80016874	T/C	1.79	1.35–2.36	0.00004	0.00022	–
		*MSH3*	rs1650737	5:80001785	A/G	1.65	1.25–2.20	0.00055	0.00184	–
		*E2F3*	rs2328524	6:20488234	G/A	1.55	1.18–2.02	0.00135	0.00857	0.20573
		*MSH3*	rs245397	5:80101773	C/T	1.69	1.23–2.32	0.00140	0.00349	–
		*E2F3*	rs10946384	6:20495546	C/T	1.70	1.23–2.36	0.00157	0.00857	–
		*E2F3*	rs4134945	6:20483603	C/T	1.85	1.27–2.70	0.00161	0.00857	–
		*E2F3*	rs2328488	6:20418329	C/T	1.49	1.12–1.99	0.00726	0.02902	–
		*LIG3*	rs3744358	17:33336914	T/G	1.45	1.10–1.92	0.00974	0.02921	0.35048
		*E2F3*	rs911361	6:20415053	G/A	0.71	0.54–0.94	0.01432	0.04581	–
		*ERCC5*	rs17655	13:103528002	G/C	0.67	0.48–0.95	0.02004	0.02004	0.32065
		*LIG3*	rs1003918	17:33332177	A/G	1.37	1.05–1.79	0.02129	0.03194	–
		*MSH3*	rs6151627	5:79965536	A/G	0.71	0.52–0.96	0.02415	0.04830	–
FL	DNA repair	*E2F2*	rs3218203	1:23837560	C/G	1.66	1.23–2.25	0.00117	0.00700	0.16809
		*APEX1*	rs1130409	14:20925154	G/T	0.68	0.52–0.88	0.00361	0.00361	0.16809
		*C11orf30*	rs1939469	11:76236220	A/G	0.57	0.37–0.87	0.00714	0.03571	0.57140
MZL	Cell death	*RELB*	rs12609547	19:45532009	G/T	2.03	1.34–3.07	0.00073	0.00145	0.05666
		*TP73*	rs1181868	1:3651126	T/G	1.99	1.31–3.01	0.00134	0.02554	0.24900
		*RELB*	rs1560725	19:45543787	T/C	1.86	1.22–2.82	0.00338	0.00338	–
		*CASP10*	rs12693932	2:202093395	T/C	1.75	1.16–2.64	0.00631	0.00631	0.12305
		*IL8RB*	rs1126579	2:219000734	T/C	0.59	0.37–0.93	0.02148	0.02148	0.24900
	Inflammation & Immunity	*LTA*	rs915654	6:31538497	T/A	0.42	0.24–0.74	0.00137	0.01097	0.32901
	DNA repair	*CHEK2*	rs5762746	22:29088123	C/T	1.87	1.25–2.78	0.00198	0.01595	0.38291
		*CHEK2*	rs1033667	22:29130300	C/T	1.83	1.22–2.73	0.00355	0.01595	–
		*YY1*	rs4905941	14:100725438	A/G	0.51	0.31–0.86	0.00761	0.01523	0.38291
		*CHEK2*	rs5762763	22:29132389	G/C	0.55	0.34–0.91	0.01518	0.04553	–
MCL	Cell death	*CASP8*	rs1035142	2:202153078	G/T	2.25	1.41–3.60	0.00056	0.00450	0.17541
		*BAK1*	rs17627049	6:33537802	C/A	0.40	0.19–0.83	0.00640	0.04478	0.58215
		*CDH22*	rs3915737	20:44871381	A/C	0.49	0.26–0.94	0.02147	0.02147	0.41874
	Inflammation & Immunity	*PRDM1*	rs6924807	6:106531266	A/G	0.44	0.25–0.76	0.00185	0.01478	0.28303
		*JAK3*	rs10419991	19:17938891	A/G	0.46	0.27–0.78	0.00270	0.01887	0.28303
		*JAK3*	rs3212760	19:17947546	A/G	0.51	0.30–0.88	0.01140	0.03989	–
	DNA repair	*LIG4*	rs1151402	13:108858030	C/T	0.40	0.23–0.69	0.00042	0.00250	0.12007
		*LIG4*	rs12428162	13:108871915	G/C	2.42	1.44–4.05	0.00084	0.00253	–
		*LIG4*	rs1805386	13:108861913	A/G	2.46	1.43–4.24	0.00156	0.00312	–
TCL	Cell death	*CASP4*	rs1944900	11:104838471	C/T	0.34	0.15–0.75	0.00199	0.01196	0.46639
		*ITCH*	rs4911154	20:32996101	G/A	2.06	1.21–3.52	0.01027	0.03082	0.47519
		*RASSF1*	rs2236947	3:50371432	C/A	1.78	1.10–2.88	0.01828	0.03655	0.47519
	Inflammation & Immunity	*IFNB1*	rs1051922	9:21077716	G/A	1.75	1.14–2.68	0.01126	0.01126	0.33787
		*IL6*	rs2069840	7:22768572	C/G	1.86	1.13–3.05	0.01421	0.04263	0.57505

* Coordinates obtained from Ensembl 64.

Testing rs33003 for association with increased risk of family history of colorectal cancer showed an association under the recessive model (OR: 0.20 [95% CI: 0.03–1.43], *p* = 0.034) but not under the additive or dominant models. The 95% confidence interval overlaps 1, however, indicating this result could be a chance finding. Furthermore, adjusting the DLBCL susceptibility analysis by family history of colorectal cancer (in addition to 5-year age group, sex, and region) did not change the OR or *p*-values of the association of rs33003 with DLBCL susceptibility. We find no evidence that family history of colorectal cancer influences the association between rs33003 and susceptibility to DLBCL.

The association of rs33003 with DLBCL did not replicate in the San Francisco sample set (OR 1.03 [95% CI: 0.83–1.29], *p* = 0.774). The minor allele frequencies of rs33003 are similar in the original population (MAF = 0.32) and the San Francisco set (MAF = 0.34). Furthermore, the *r* ^2^ value between rs33003 and rs181747 is similar in the two populations (*r* ^2^ = 0.65 in the original population and *r* ^2^ = 0.67 in the San Francisco population). This indicates that the failure to replicate is unlikely to be due to population-specific differences in minor allele frequencies or LD structure in that area of the genome.

One other SNP, rs12609547, in *RELB*, was mildly associated with marginal zone lymphoma (OR: 2.03 [95% CI: 1.34–3.07], *p*_G_ = 0.0015). This association was not significant, however, after multiple testing correction at the hypothesis level (*p*_H_ = 0.0570).

## Discussion

After multiple testing correction within genes, there was evidence for associations of NHL subtypes with SNPs in two genes: *RELB* with MZL and *MSH3* with DLBCL. Only the *MSH3* association, however, was significant after the additional correction for multiple testing between genes. This association, however, did not replicate in another North American population [15], indicating that it was likely a type I error.

MSH3 is involved in DNA mismatch repair (MMR), which corrects mismatched or unmatched bases and small insertion/deletion loops that result from DNA replication before cell division or from DNA repair processes [17]. The MMR pathway is an important repair mechanism in normal lymphocyte development as evidenced by mouse models and human patients deficient in this pathway [18]. Studies of MMR deficiency and MMR gene deregulation in lymphomas have also illustrated the potential role of this pathway in NHL[19]–[23].

Because of the MMR pathway’s established role in hereditary non-polyposis colorectal cancer (HNPCC), we tested whether rs33003 was associated with a family history of colorectal cancer in first degree relatives. Adjusting the DLBCL susceptibility analysis by family history of colorectal cancer in addition to 5-year age group, sex, and region did not change the analysis results, indicating that family history of colorectal cancer is not a confounder for susceptibility to DLBCL. Furthermore, we did not find that rs33003 was associated with a family history of colorectal cancer. This is not entirely surprising, as colorectal cancer is not associated with lymphoma [24], although mismatch repair cancer syndrome is characterized in part by a combination of colorectal polyposis, [25] and early-onset hematologic cancers [26], [27].

DLBCL can be subdivided into at least three subgroups using molecular signatures [28]. It is therefore possible that the *MSH3* association is confined to patients with tumours belonging to specific DLBCL subgroups. We do not, however, have molecular signature data for the tumours of the DLBCL patients included in this study. It is also possible that there are true associations with NHL susceptibility that we are not able to detect in this study. This could be due to low sample sizes for some subtypes of NHL in our study, or perhaps population-specific effects. This could explain our inability to replicate candidate gene (**Table 1**) associations of SNPs in *IRF4* with FL [8], or our observation of weak associations (i.e. a SNP with *p*<0.05 but that does not pass multiple testing correction) of SNPs in *BID*, *APAF1* and *CASP10* with NHL [2]. We were also unable to replicate other associations for SNPs in the “replication” category, listed in **Table S2 in File S1**. Furthermore, HapMap coverage may not have been adequately deep to represent causal variants present in some genes we assayed, making our tagSNP approach vulnerable to false negative results. As in most other lymphoma studies[1]–[8], multiple testing correction was not done for the number of subtypes tested as the subtypes are considered separate disease entities, with different presentation, possible etiology and hypotheses. Finally, any association reported here could be an association with survival as opposed to susceptibility, as patients who have less aggressive disease are more likely to have time to participate in the study and provide a DNA sample. This is not likely, however, given the low percentage of cases who died prior to contact (10.5% in the British Columbia study [10] and 14.2% in the San Francisco set [15]).

In summary, we found no replicated associations in the genes studied related to immunity and inflammation, DNA repair and programmed cell death.

## Supporting Information

File S1 **Table S1.** Candidate genes chosen based on biological interest. **Table S2.** SNPs tested for replication. **Table S3.** SNPs that passed quality control. **Table S4.** Logistic regression analysis results for SNPs with ***p_G_***<0.05 before multiple testing correction. **Table S5.** The best model for rs33003 in European-ancestry DLBCL vs. controls is the recessive model.
(XLS)
